# *Mycobacterium bovis* Strain-Dependent Effects of ESAT-6 and CFP-10 on Inflammasome Activation in Bovine Macrophages

**DOI:** 10.3390/ijms27094099

**Published:** 2026-05-03

**Authors:** Federico Carlos Blanco, Cristina Lourdes Vazquez, María Mercedes Bigi, Rosana Valeria Rocha, Elizabeth Andrea García, Fabiana Bigi

**Affiliations:** 1 Instituto de Agrobiotecnología y Biología Molecular (IABIMO), INTA-CONICET, Hurlingham 1686, Argentina; 2 Instituto de Biotecnología, CICVyA, Instituto Nacional de Tecnología Agropecuaria, N. Repetto and De los Reseros, Hurlingham 1686, Argentina; 3 Instituto de Investigaciones Biomédicas (INBIOMED), CONICET-Universidad de Buenos Aires, Buenos Aires C1121ABG, Argentina

**Keywords:** *Mycobacterium bovis*, ESAT-6, inflammasome, bovine macrophage, IL-1β

## Abstract

*Mycobacterium bovis*, the causative agent of bovine tuberculosis, infects and persists within macrophages, triggering pro-inflammatory responses. While these mechanisms are well characterized for *Mycobacterium tuberculosis*, less is known about host responses to *M. bovis*. Inflammasome activation and IL-1β production have been linked to ESAT-6, a substrate of the ESX-1 secretion system present in both species. Here, we examined inflammasome activation in bovine macrophages infected with the virulent *M. bovis* strain Mb04-303. *M. bovis* AF2122/97 and NCTC10772 upregulated IL-1β transcription, whereas Mb04-303 and BCG did not. Unexpectedly, deletion of the genes encoding ESAT-6 and CFP-10 from Mb04-303 enhanced inflammasome activation, as evidenced by increased NLRP3 and IL-1β transcription. Complementation with either wild-type ESAT-6/CFP-10 or the T63A ESAT-6 variant restored downregulation of the response, indicating that this substitution does not alter inflammasome modulation. In contrast, deletion of ESAT-6/CFP-10 from an attenuated *M. bovis* vaccine candidate reduced IL-1β transcription. No differences were observed between *M. tuberculosis* H37Rv and its ESAT-6-deficient mutant in bovine macrophages. Together, these findings demonstrate that ESAT-6/CFP-10-mediated modulation of inflammasome activation in bovine macrophages is highly dependent on the mycobacterial genetic background.

## 1. Introduction

*Mycobacterium bovis*, a key member of the *Mycobacterium tuberculosis* complex (MTBC), is the primary etiological agent of bovine tuberculosis (bTB). This chronic infectious disease represents a significant threat to cattle populations and exhibits considerable zoonotic potential, affecting a wide range of mammalian hosts, including humans [1]. Zoonotic transmission to humans occurs predominantly through two routes: occupational exposure in agricultural settings and consumption of unpasteurized dairy products derived from infected animals [1]. Beyond the public health implications, bTB imposes a substantial economic burden arising from direct losses in livestock productivity and carcass condemnation, as well as indirect costs associated with international trade restrictions on affected meat and dairy products.

One of the most promising strategies for reducing the prevalence of bTB is vaccination. The Bacillus Calmette–Guérin (BCG) vaccine, currently the only licensed vaccine against human tuberculosis, has demonstrated efficacy in reducing bTB transmission in livestock [2]. However, the protection conferred by BCG is partial and variable [3]. Consequently, the development of an effective vaccine against bTB remains a critical goal, which requires a deeper understanding of the interactions between *M. bovis* and its host.

Inflammasomes are intracellular multiprotein complexes that function as critical sensors of infection and cellular stress [4]. These complexes, typically composed of a sensor protein (e.g., NLRP3 or AIM2), the adaptor protein ASC (apoptosis-associated speck-like protein containing a CARD), and the effector caspase-1, mediate a robust inflammatory response upon activation [5]. This pathway drives the proteolytic maturation and secretion of potent proinflammatory cytokines, most notably interleukin-1β (IL-1β), which plays a central role in antimicrobial host defense [6,7,8]. During infection with pathogenic mycobacteria, inflammasome activation in macrophages is thought to be initiated when bacterial components breach the phagosomal membrane and are detected by cytosolic pattern recognition receptors.

Caspase-1 activation during *M. tuberculosis* infection depends on live intracellular bacilli expressing a functional ESX-1 secretion system encoded within the region of difference 1 (RD1), which is absent from the vaccine strain *M. bovis* BCG [9]. The ESX-1 system mediates the secretion of the immunodominant virulence factors ESAT-6 (EsxA) and CFP-10 (EsxB), which play key roles in host–pathogen interactions. ESAT-6 contributes directly to inflammasome activation by promoting phagosomal membrane damage and facilitating the cytosolic translocation of mycobacterial products, including Ag85, thereby enabling inflammasome sensing [9,10]. As a result, *M. tuberculosis* infection predominantly activates an NLRP3- and ASC-dependent inflammasome complex in macrophages, leading to caspase-1 activation, IL-1β maturation, and host cell death through mechanisms that include necrosis [11,12,13]. Although most studies investigating the role of ESAT-6 in host cell modulation rely on recombinant protein, one notable exception demonstrated that an *M. tuberculosis* mutant lacking *esxA* exhibits impaired IL-1β secretion, directly implicating ESAT-6 as an activator of the NLRP3/ASC inflammasome [9].

In addition to its involvement in innate immunity, recombinant ESAT-6 induces apoptosis in THP-1 cells and inhibits autophagic flux in RAW264.7 cells and primary murine peritoneal macrophages [14,15]. ESAT-6 also elicits strong adaptive immune responses in both cattle and humans [16,17,18], a property that has been widely exploited in tuberculosis diagnosis and vaccine development [19].

We have previously characterized the innate immune response elicited by infection with *M. bovis* Mb04-303, which was isolated from a wild boar [20,21]. We demonstrated that Mb04-303 induces a markedly stronger innate immune response in bovine macrophages than the closely related strain Mb534. Infection with Mb04-303 was associated with robust activation of the type I interferon signaling cascade, whereas the KEAP1–NFE2L2 axis—known to regulate antioxidant gene expression and contribute to inflammasome-related responses—was comparatively suppressed relative to Mb534. Furthermore, macrophages infected with Mb04-303 exhibited increased recruitment of Galectin-8, consistent with enhanced phagosomal membrane damage. This observation suggests greater exposure of bacterial components to the cytosol, potentially facilitating activation of cytosolic sensing pathways that drive type I interferon responses [22].

On the other hand, analysis of the *M. bovis* genome database revealed that ESAT-6 is highly conserved across the species, except for a single non-synonymous mutation in ESAT-6 of the Mb04-303 strain [23]. ESAT-6 and CFP-10 are known to form a tight 1:1 heterodimeric complex, in which both proteins adopt fully folded structures [24]. We further demonstrated that ESAT-6 can self-interact using an *Escherichia coli* two-hybrid system. Notably, the ESAT-6 allele from Mb04-303 showed a stronger interaction than the wild-type allele [21].

In a previous study, we generated an Mb04-303 mutant lacking the *esxA* and *esxB* genes. Given the established role of ESAT-6 in regulating intracellular trafficking and inflammasome activation in infected macrophages, we hypothesized that deletion of these ESX-1 substrates would significantly alter inflammasome activation in bovine macrophages. Therefore, the objective of the present study was to use this previously constructed mutant to define the contribution of ESAT-6 and CFP-10 to inflammasome activation and IL-1β transcription in the natural host, thereby clarifying their role in shaping the innate immune response to *M. bovis* infection.

## 2. Results

### 2.1. M. bovis Strains Differ in Their Capacity to Trigger Inflammasome Activation

**Figure 1:**
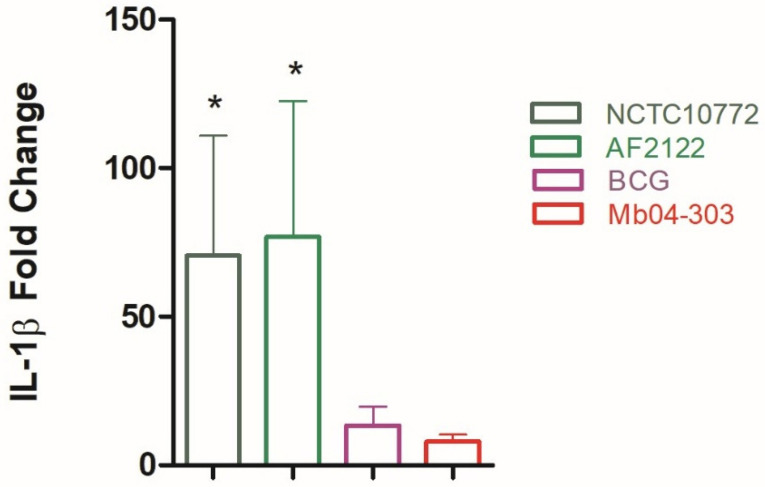
Relative expression of IL-1β in bovine macrophages purified from 6 to 7 cattle and infected with NCTC10772, AF2122, BCG or Mb04-303. Macrophages were infected at a multiplicity of infection (MOI) of 1:1. IL-1β transcript levels were quantified by real-time RT-PCR using GAPDH as an endogenous reference gene and uninfected cells as the calibrator. Data are presented as mean ± SEM. The pairwise fixed reallocation randomization test was used to assess differences between each infected group and the uninfected control (* *p* < 0.05).

We first assessed inflammasome activation by measuring IL-1β transcript levels in bovine macrophages infected with different *M. bovis* strains. IL-1β transcription was evaluated in macrophages infected with the reference strain AF2122/97, NCTC10772, the Mb04-303 strain, and the vaccine strain BCG. As shown in Figure 1, AF2122/97 and NCTC10772 induced significantly higher levels of IL-1β gene expression relative to uninfected macrophages. As expected, BCG did not induce IL-1β expression. Unexpectedly, Mb04-303 also failed to upregulate IL-1β, despite previous studies identifying this strain as more virulent than other *M. bovis* isolates [25]. This finding is consistent with studies on inflammasome activation in clinical isolates of *M. tuberculosis*, in which more virulent isolates exhibit lower IL-1β levels [26].

### 2.2. Inflammasome Activation in Macrophages Infected with Mb04-303 and Mb04-303ΔesxAB

**Figure 2:**
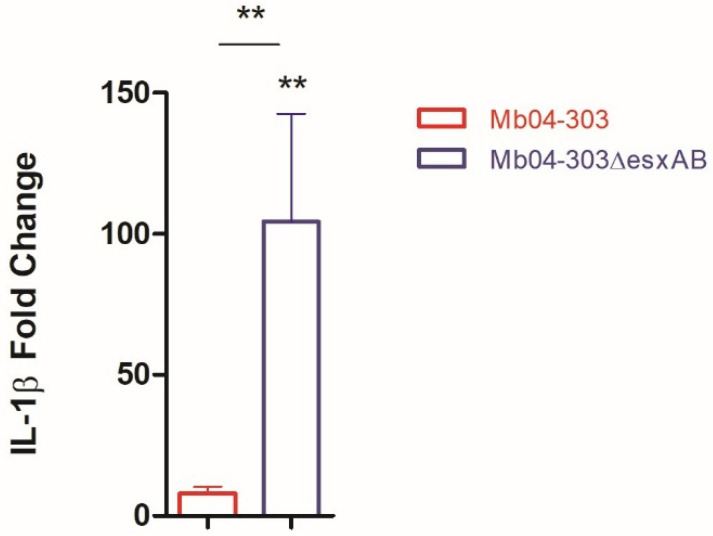
Relative expression of IL-1β in bovine macrophages purified from 6 to 7 cattle and infected with Mb04-303 or Mb04-303Δ*esxAB*. Macrophages were infected at a multiplicity of infection (MOI) of 1:1. IL-1β transcript levels were quantified by real-time RT-PCR using GAPDH as an endogenous reference gene and uninfected cells as the calibrator. Values represent the mean ± SEM. Statistical significance was evaluated using Kruskal–Wallis test (** *p* < 0.01). The pairwise fixed reallocation randomization test was used to assess differences between each infected group and the uninfected control (** *p* < 0.01).

One distinctive feature of Mb04-303 is the T63A non-synonymous mutation in the *esxA* gene [23], which encodes ESAT-6, an ESX-1 substrate implicated in inflammasome activation. Based on this, we investigated the contribution of ESAT-6 to inflammasome activation. A mutant strain lacking *esxA* and *esxB* (the latter encoding CFP-10) induced a significant upregulation of IL-1β transcript levels in infected bovine macrophages compared to both uninfected cells and the parental Mb04-303 strain (Figure 2). These results suggest that, in the genomic background of Mb04-303, ESAT-6 may exert a regulatory—or even opposing—effect on inflammasome activation, in contrast to its previously described pro-inflammatory role in *M. tuberculosis.*

### 2.3. Impact of the ESAT-6 T63A Mutation on CFP-10 Binding and Inflammasome Activation

The identification of a non-synonymous mutation in *esxA* (T63A) in Mb04-303 led us to examine its potential effects on the structural stability and interaction dynamics of the ESAT-6/CFP-10 heterodimer. Structural analyses were conducted using the high-resolution crystal structure of the CFP-10/ESAT-6 complex from *M. tuberculosis* (PDB ID: 3FAV). The ESAT-6 sequence in this structure is identical (100%) to the wild-type ESAT-6 analyzed in this study, ensuring direct structural correspondence.

**Figure 3:**
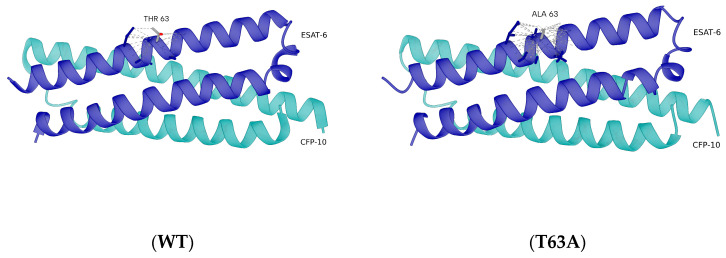
Structural representation of the ESAT-6/CFP-10 heterodimer based on the crystal structure (PDB ID: 3FAV) from *M. tuberculosis*. The structure comprises the experimentally resolved fragments of ESAT-6 (*esxA*, residues 2–95) and CFP-10 (*esxB*, residues 1–100). The ESAT-6 sequence is 100% identical to the corresponding protein in *M. bovis*, allowing direct structural interpretation for the studied strain. Residue 63 is shown highlighted in both the wild-type and T63A models, illustrating its surface localization outside the ESAT-6/CFP-10 interaction interface. WT: wild type, T63A: mutant.

Mapping of residue 63 onto the three-dimensional structure showed that it is surface-exposed and located outside the ESAT-6/CFP-10 interaction interface (Figure 3). Structural superposition of the wild-type and T63A ESAT-6 models revealed highly similar backbone conformations, with a root mean square deviation (RMSD) of approximately 0.1 Å calculated over Cα atoms, indicating that the T63A substitution does not induce global structural rearrangements (Figure S1). Short-range interprotein contacts between ESAT-6 and CFP-10 were assessed using a conservative 4 Å distance cutoff, demonstrating that residue 63 maintains a comparable local contact environment in both models, with no detectable loss of interfacial contacts upon mutation.

In silico predictions using multiple computational tools consistently supported a modest and non-disruptive effect of the T63A substitution (Table S1). MutaBind2 predicted a near-neutral effect on protein–protein interaction free energy (ΔΔG_bind = 0.01 kcal/mol) and classified residue 63 as non-interfacial. FoldX (via MutaBind2) estimated a slight stabilization of the mutant within the heterodimer (ΔΔG_fold = −0.29 kcal/mol), whereas I-Mutant 2.0 predicted a moderate decrease in intrinsic stability of monomer ESAT-6 (ΔΔG = −0.85 kcal/mol). Although numerical estimates differed among predictors, these differences reflect methodological variations and the distinct structural contexts evaluated (heterodimer vs. monomer). Importantly, all methods converged on a consistent interpretation of a minor, non-disruptive structural impact.

**Figure 4:**
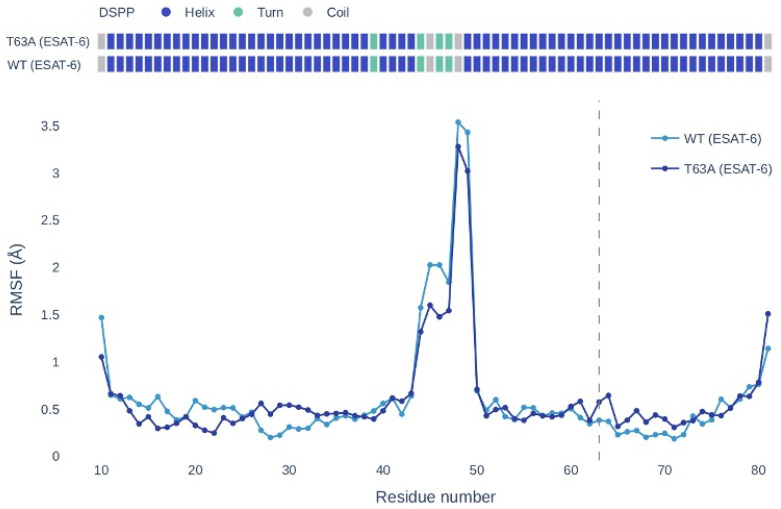
Residue-wise root mean square fluctuation (RMSF) profiles of the ESAT-6 chain obtained from CABS-flex simulations for the wild-type (WT) and T63A mutant complexes. The T63A substitution induces a localized increase in flexibility around residue 63 (dashed line) and neighboring residues, accompanied by a subtle redistribution of fluctuation amplitudes in distal regions. No evidence of global destabilization or emergence of alternative conformational states was observed, consistent with preservation of the overall dynamic profile of the ESAT-6/CFP-10 heterodimer.

To move beyond static structural analyses, we performed coarse-grained flexibility simulations using CABS-flex. Comparison of wild-type and T63A conformational ensembles revealed a localized, low-amplitude increase in flexibility around residue 63, without evidence of major alterations in the global conformational behavior of the heterodimer (Figure 4). Quantitative comparison of RMSF profiles showed that the increase in flexibility at residue 63 and neighboring positions (B61-B65) was modest (ΔRMSF ≈ 0.1–0.2), whereas distal regions exhibited virtually identical fluctuation patterns between wild-type and mutant complexes.

**Figure 5:**
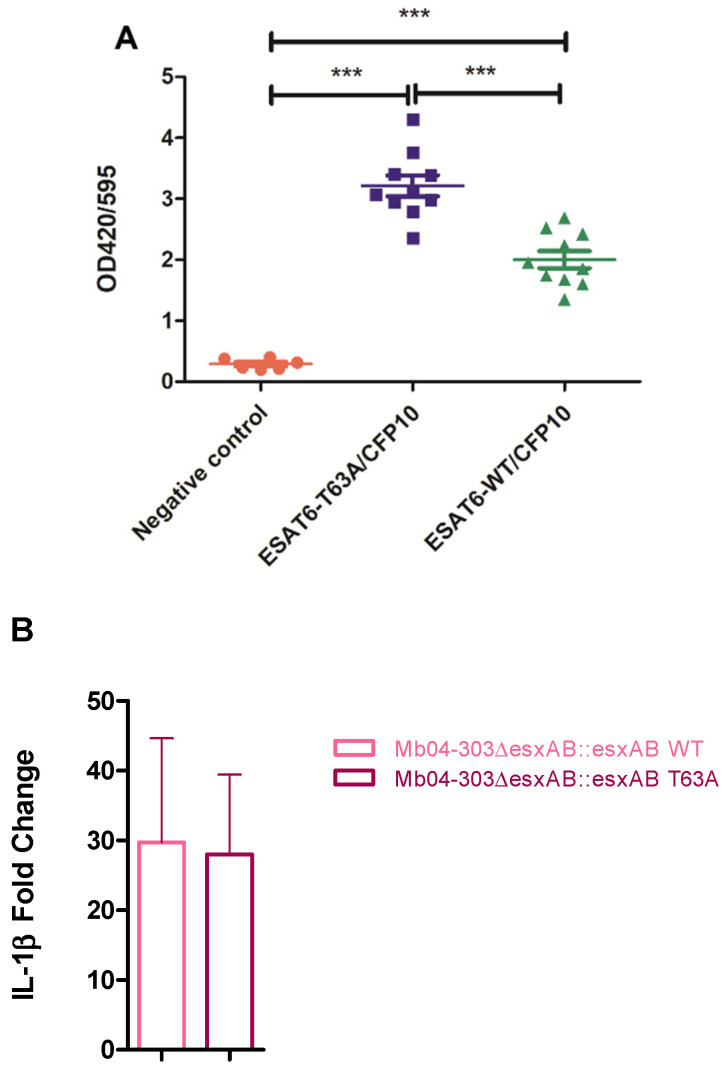
Role of the T63A mutation in ESAT-6 on CFP-10 interaction and inflammasome activation. (**A**) Interaction between ESTA6 and CFP10. β-galactosidase activity was expressed per microgram of total protein in *E. coli* BTH101 co-transformed with T25-ESAT6 and T18-CFP10. Similar results were obtained using the reciprocal construct combination (T18-ESAT6 and T25-CFP10). Data represent the mean of ten independent cultures. Statistical significance was assessed using ANOVA and Tukey’s multiple comparison test (*** *p* < 0.001). (**B**) Relative expression of IL-1β mRNA in bovine macrophages purified from 6 to 7 cattle and infected with the complemented strains Mb04-303Δ*esxAB*::*esxAB* WT or Mb04-303Δ*esxAB*::*esxAB* T63A. Macrophages were infected at a multiplicity of infection (MOI) of 1:1. IL-1β transcript levels were quantified by real-time RT-PCR using GAPDH as an endogenous reference gene and uninfected cells as the calibrator. Data are presented as mean ± SEM. Statistical significance was assessed using the Kruskal–Wallis test and a randomization test to evaluate differences between strains and the uninfected control, respectively (*p* > 0.05).

Next, we assessed the interaction between ESAT-6 and CFP-10 using a two-hybrid system. In previous studies, we demonstrated that the self-interaction of the T63A ESAT-6 mutant was stronger compared to the wild-type ESAT-6 [21]. Consistent with these observations, the interaction between CFP-10 and the T63A ESAT-6 variant was stronger than that with the wild-type ESAT-6 (Figure 5A). Of note, the increased signal observed in the two-hybrid system may reflect effects of the fusion proteins on stability or solubility rather than enhanced native interaction, consistent with the neutral predictions from structural analyses.

To further assess the impact of the T63A mutation on inflammasome activation in infected macrophages, we evaluated IL-1β transcript levels in the deletion mutant complemented with either the T63A variant or the wild-type ESAT-6 allele, both co-expressed with CFP-10. IL-1β transcript levels were not significantly upregulated in macrophages infected with either complemented strains compared to uninfected cells (Figure 5B), indicating that the T63A substitution in ESAT-6 does not account for the observed downregulation of the inflammasome response. Moreover, complementation restored the Mb04-303 wild-type phenotype (i.e., no significant inflammasome activation). Collectively, these structural, dynamic, and functional results support the interpretation of T63A as a structurally tolerated surface substitution that does not seem to impair ESAT-6/CFP-10 function.

### 2.4. Activation of the Inflammasome Is Mediated by the NLRP3 Sensor

**Figure 6:**
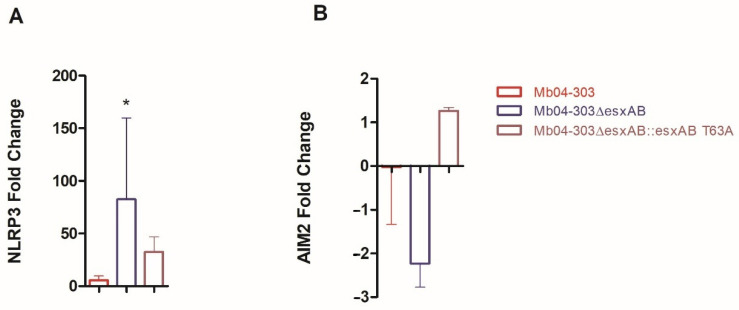
Relative expression of NLRP3 (**A**) and AIM2 (**B**) in Mb04-303, Mb04-303Δ*esxAB* and Mb04-303Δ*esxAB*::*esxAB* T63A infected macrophages. Cells were isolated from 6 to 7 cattle and infected at a multiplicity of infection (MOI) of 1:1. Transcript levels were quantified by real-time RT-PCR using GAPDH as an endogenous reference gene and uninfected cells as calibrator. Data are expressed as mean ± SEM. Differences between each infected group and the uninfected control were assessed using the pairwise fixed reallocation randomization test (* *p* < 0.05).

Previous studies on inflammasome activation in cellular infection contexts have identified two main mechanisms, primarily mediated by NLRP3 or AIM2, among others [7]. To elucidate the activation pathway responsible for the increased IL-1β in macrophages infected with the Mb04-303, Mb04-303Δ*esxAB*, and complemented strains, we analyzed the transcript levels of both mediators. Notably, only cells infected with Mb04-303Δ*esxAB* exhibited increased NLRP3 transcript levels relative to the uninfected control, which coincided with the highest IL-1β expression (Figure 6A). No significant elevation of NLRP3 levels was observed following infection with the other strains. In the case of AIM2, no significant changes in transcript levels were detected under any of the experimental conditions analyzed (Figure 6B). Taken together, these findings suggest that inflammasome activation in the strains examined is predominantly mediated by NLRP3.

IL-18 is a cytokine generated following inflammasome activation [27]. Accordingly, we assessed its transcript levels across the different strains and found no increase in IL-18 transcript levels in the Mb04-303 mutant strain (Figure S2).

**Figure 7:**
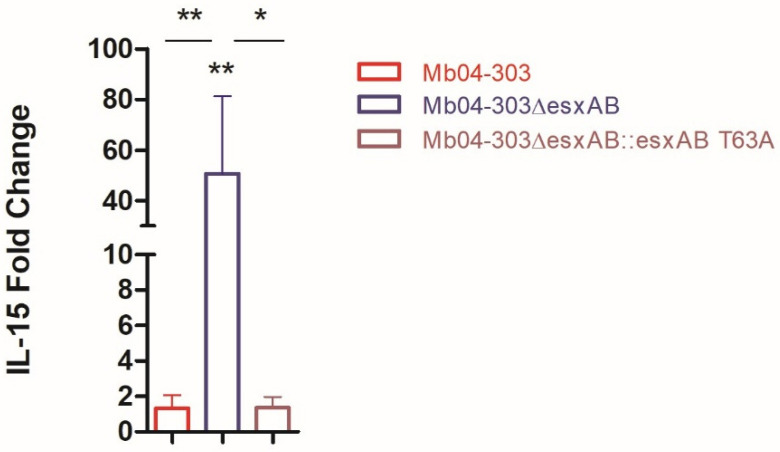
Relative expression of IL-15 in Mb04-303, Mb04-303Δ*esxAB* and Mb04-303Δ*esxAB*::*esxAB* T63A infected macrophages. Macrophages were isolated from 6 to 7 cattle and infected at a multiplicity of infection (MOI) of 1:1. Transcript levels were quantified by real-time RT-PCR using GAPDH as an endogenous reference gene and uninfected cells as the calibrator. Values represent the mean ± SEM. Statistical significance was evaluated using Kruskal–Wallis test followed by Dunn’s post hoc test for multiple comparisons (* *p* < 0.05; ** *p* < 0.01). The pairwise fixed reallocation randomization test was used to assess differences between each infected group and the uninfected control (** *p* < 0.01).

Given that IL-15 activates NF-κB signaling and that NF-κB is central to inflammasome priming, we also assessed the effect of ESAT-6/CFP-10 deletion on IL-15 transcription in *M. bovis*–infected macrophages. IL-15 transcription exhibited a pattern comparable to that observed for NLRP3 and IL-1β (Figure 7).

### 2.5. Deletion of esxA-B Does Not Affect PDIM Levels or Composition

PDIM synthesis and proper transport are essential for the coordinated release of ESX-1 substrates, phagosomal membrane disruption, and subsequent activation of type I interferon signaling pathways [28]. Considering these previous findings, we explored whether deletion of *esxA-B* in the Mb04-303 strain may impact PDIM content and, in turn, modulate inflammasome activation in infected macrophages. Figure S3 shows that PDIM levels were comparable among the wild-type, mutant, and complemented strains, indicating that the differential phenotypes observed with respect to inflammasome-related gene expression are unlikely to be attributable to differences in PDIM quantity or composition.

### 2.6. ESAT-6/CFP-10 Deletion in Pathogenic Mycobacteria Affects Inflammasome Activation

Previous studies have linked inflammasome activation to phagosomal escape, a process directly associated with ESAT-6 [29]. Given our unexpected results with the Mb04-303Δ*esxAB* mutant, we further investigated IL-1β responses using two additional ESAT-6 deletion mutants, one in *M. bovis* and one in *M. tuberculosis*.

**Figure 8:**
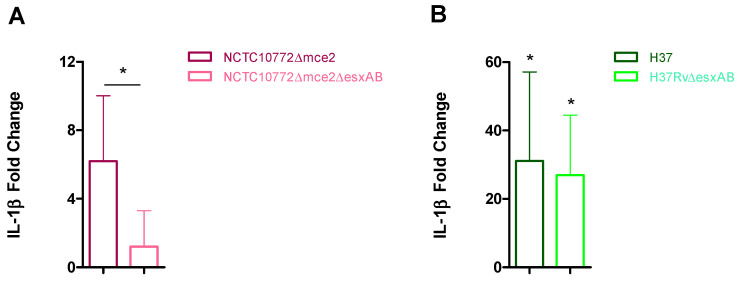
Relative expression of IL-1β in NCTC10772Δ*mce2AB* and NCTC10772Δ*mce2AB*Δ*esxAB* (**A**) H37Rv and H37RvΔ*esxAB* (**B**) infected macrophages. Samples were obtained from 6 to 7 cattle and infected at a multiplicity of infection (MOI) of 1:1. Transcript levels were quantified by real-time RT-PCR using *GAPDH* as an endogenous reference gene and uninfected cells as the calibrator. Values represent the mean ± SEM. Statistical significance was evaluated using Mann–Whitney unpaired test (*p* < 0.05). The pairwise fixed reallocation randomization test was used to assess differences between each infected group and the uninfected control (* *p* < 0.05).

We previously generated an *esxA–esxB* deletion in the MbΔ*mce2AB* background [30]. This double mutant (MbΔ*mce2AB*Δ*esxAB*), together with its parental strain (MbΔ*mce2AB*), was evaluated for its ability to induce IL-1β transcript levels in bovine macrophages. As shown in Figure 8A, MbΔ*mce2AB*Δ*esxAB* induced lower IL-1β transcript levels than its parental strain.

In parallel, we compared IL-1β transcript levels in macrophages infected with the *M. tuberculosis* strain H37Rv and its corresponding H37RvΔ*esxA* mutant (kindly provided by the Sherman laboratory). As shown in Figure 8B, both strains significantly upregulated IL-1β transcript levels compared to the uninfected control; however, the difference in fold change between the two strains was not significant. Collectively, these results underscore the critical influence of the mycobacterial genomic background and the experimental context in determining inflammasome activation.

## 3. Discussion

Inflammasome activation by *M. bovis* remains significantly less well characterized than that induced by *M. tuberculosis*, particularly in macrophages derived from the natural bovine host. A recent study using the *M. bovis* AN5 strain and culture filtrate extract reported that inflammasome activation in bovine macrophages occurs predominantly through the NLRP3 pathway [12], consistent with our observations. Additional research examining mitochondrial damage during *M. bovis* infection has supported a subordinate role for AIM2 relative to NLRP3 [31]. In contrast, studies performed in human THP-1 cells using Beijing lineage strains of *M. bovis* implicated both NLRP3 and NLRP7 in inflammasome activation [32]. Conversely, a functional contribution of AIM2 to IL-1β production has been described in murine macrophages infected with *M. bovis* [33], highlighting variability in inflammasome sensor usage depending on the host species and experimental context.

Comparative analyses further demonstrated that BCG engages inflammasome pathways inefficiently, as evidenced by limited transcriptional induction of inflammasome-related genes and modest IL-1β secretion [34], findings that are consistent with our comparison between BCG and virulent *M. bovis* isolates. However, most of these studies were conducted in murine macrophages, limiting host-specific interpretation.

By employing primary bovine macrophages and a panel of *M. bovis* strains with differential virulence, including BCG, our study provides host-relevant insights into strain-specific inflammatory outcomes. We demonstrate that deletion of *esxA* and *esxB* from the *M. bovis* isolate Mb04-303 results in enhanced IL-1β transcript levels in bovine macrophages. These findings indicate that, in this genetic background, ESAT-6 alone or the ESAT-6/CFP-10 complex exerts an inhibitory effect on inflammasome activation. The increased proinflammatory response observed upon infection with the Mb04-303 Δ*esxAB* mutant was associated with upregulation of NLRP3 transcript levels, suggesting involvement of the NLRP3 inflammasome pathway.

Comparative in silico analyses of the mutant and wild-type alleles of ESAT-6 did not detect a substantial impact of the T63A substitution on its interaction with CFP-10. These analyses require careful interpretation, particularly given the variability observed across predictive tools. FoldX (via Mu-taBind2) estimated a marginal stabilizing effect (ΔΔG_fold = −0.29 kcal/mol) when evaluating the mutation in the context of the full ESAT-6/CFP-10 heterodimer, whereas I-Mutant 2.0 predicted a modest decrease in stability (ΔΔG = −0.85 kcal/mol) when analyzing ESAT-6 as an isolated monomer. These differences likely arise from the distinct structural contexts evaluated by each tool, as well as to differences in their underlying methodologies and training datasets. Importantly, although ΔΔG definitions differ between predictors and are not directly comparable in sign, both indicate a low-magnitude effect within the range typically associated with structurally tolerated substitutions. This interpretation is further supported by the low conservation score at position 63 (PremPS: PSSM = 0.0427), consistent with a surface-exposed residue under limited functional constraint.

Given the inherent limitations of static structural predictors, we complemented this analysis with coarse-grained molecular dynamics simulations (CABS-flex) to assess the potential impact on protein flexibility. These simulations revealed a localized, low-amplitude increase in flexibility around residue 63, without detectable changes in the global conformational ensemble of the heterodimer.

These computational results contrast with the stronger ESAT-6/CFP-10 interaction observed for the T63A variant in the two-hybrid assay compared to the wild-type allele. One possible explanation is that, in this system, ESAT-6 and CFP-10 are expressed as fusion proteins with adenylate cyclase subunits [35], which may not fully preserve their native conformation. In this context, the T63A substitution could enhance the apparent interaction between bait and prey proteins by increasing the stability of the fusion constructs—an effect that may not occur in the native proteins. Consistent with the in silico results, the T63A substitution in ESAT-6 from Mb04-303 did not influence IL-1β transcript levels, indicating that this polymorphism does not account for the observed phenotype.

Because the results of this study contrast with previous reports describing a role for ESAT-6 in promoting inflammasome activation during *M. tuberculosis* infection, it is important to consider the extensive body of evidence supporting a proinflammatory function of ESX-1 in this pathogen. Previous studies have demonstrated that IL-1β production during *M. tuberculosis* infection depends on activation of the NLRP3 inflammasome and a functional ESX-1 secretion system. Infection of THP-1 macrophages with live *M. tuberculosis* induces caspase-1 activation and IL-1β secretion in an ESX-1-dependent manner requiring ESAT-6 expression [9]. ESAT-6 has been shown to be both necessary and sufficient to trigger caspase-1 activation, primarily by promoting phagosomal membrane damage and facilitating the translocation of mycobacterial components into the cytosol.

ESAT-6–mediated membrane perturbation has also been linked to necrotic and pyroptotic cell death, and mutants lacking *esxA* failed to induce necrosis and produced significantly lower levels of IL-1β in differentiated THP-1 macrophages and primary human macrophages [36]. Furthermore, ESAT-6–dependent activation of NLRP3 requires Syk tyrosine kinase signaling, linking phagosomal disruption to inflammasome assembly [36]. Consistently, bone marrow–derived macrophages infected with an H37Rv *esxA* mutant generated reduced levels of mature IL-1β compared with cells infected with wild-type H37Rv, despite comparable induction of pro-IL-1β [37]. In vivo, intranasal delivery of ESAT-6, but not CFP-10, induced pulmonary IL-1β expression together with elevated serum amyloid A3 (SAA3), which seems critical for optimal IL-1β production by BMDMs [38].

In summary, ESX-1–mediated membrane damage has been associated with potassium efflux, NLRP3 activation, gasdermin D–dependent pyroptosis, and IL-1β release, with additional contributions from mitochondrial signaling pathways that facilitate inflammasome assembly [37].

To address the discrepancy between our findings in Mb04-303 and the established proinflammatory role of ESAT-6 in *M. tuberculosis*, we evaluated IL-1β transcript levels in an *esxAB* mutant generated in a different genomic background. Using a previously developed vaccine candidate harboring deletions in *mce2AB* and *esxAB*, we observed reduced IL-1β transcript levels in macrophages infected with the double mutant compared with the *mce2AB* single mutant, supporting a role for ESAT-6 or ESAT-6/CFP-10 dimmer in enhancing inflammasome activation in that context.

Deletion of *mce1* has previously been shown to modulate IL-1β expression in murine macrophages infected with *M. tuberculosis* [39]. More recently, Mce2D, a component of the Mce2 transporter [40], was reported to block M1 polarization in THP1 macrophages, which resulted in diminished IL-1β expression [41]. These findings are consistent with our study, in which IL-1β transcript levels in the MbΔ*mce2AB* mutant (mean fold change: 6.19) were similar to those observed in Mb04-303 (7.91) and BCG (13.48), while an even more pronounced reduction was observed in the MbΔ*mce2AB*Δ*esxAB* mutant.

In addition, IL-1β transcript levels were upregulated in bovine macrophages infected with H37Rv. However, deletion of *esxA* in this strain did not alter inflammasome activation, further supporting the idea that the contribution of ESAT-6 to the proinflammatory response is influenced by the strain’s genomic background and the specific in vitro context.

One limitation of this study is that our findings do not fully elucidate the molecular mechanism by which ESAT-6/CFP-10 attenuates inflammasome activation under our experimental conditions. However, based on our previous research, we propose a plausible mechanistic explanation. We have previously demonstrated that infection of bovine macrophages with Mb04-303 induces marked modulation of innate immune pathways, characterized by downregulation of the KEAP1–NFE2L2 axis—an important regulatory pathway linked to redox balance and inflammasome control—and activation of type I interferon (IFN-I) signaling [22]. In this context, ESAT-6/CFP-10 may contribute to reshaping the macrophage response by simultaneously dampening KEAP1–NFE2L2–dependent regulation and enhancing IFN-I responses, thereby creating a cellular environment less permissive to inflammasome activation.

Additionally, ESAT-6 may indirectly modulate inflammasome activity through its capacity to promote IFN-γ production by natural killer (NK) cells present in macrophage cultures. We have also shown that Mb04-303 elicits robust IFN-γ production by NK cells, at least partially dependent on ESAT-6 expression [21]. Notably, IFN-γ produced by lymphocytes during *M. tuberculosis* infection has been reported to inhibit inflammasome activation under certain conditions. Therefore, ESAT-6/CFP-10 could suppress inflammasome responses both directly—via modulation of macrophage intrinsic pathways—and indirectly—through cytokine-mediated crosstalk with NK cells.

## 4. Materials and Methods

### 4.1. Mycobacterial Strains

Mb04-303Δ*esxAB* was generated using a mycobacteriophage-mediated transduction method [42], in which the *esxA* and *esxB* genes were replaced with a kanamycin resistance cassette. Complemented strains were constructed by PCR amplification of the *esxA–esxB* operon from Mb04-303 or NCTC10772 (using primers 5′CATATGACAGAGCAGCAGTG 3′and 5′AAGCTTTCAGAAGCCCATTTGCG 3′), followed by cloning into the *NdeI* and *HindIII* restriction sites of the replicative plasmid pVV16. MbΔ*mce2AB* and MbΔ*mce2AB*Δ*esxAB* were previously obtained. First an unmarked-chromosomal deletion of the region spanning the *mce2A*-*mce2B* genes was generated in NCTC10772 strain [30] and then by mycobacterial recombineering method the genes *esxA* and *esxB* were deleted from MbΔmce2*AB* [30].

### 4.2. Mycobacterial Growth Conditions

Mb04-303 and NCTC10772 wild-type strains, as well as their respective derivative strains, were grown in Middlebrook 7H9 broth or on 7H10 agar supplemented with 0.4% sodium pyruvate, 0.5% bovine serum albumin, 0.4% glucose, and 0.05% Tween 80. The AF2122/97 strain was cultured under the same conditions with the addition of 0.2% glycerol. BCG, H37Rv, and its derivative strain were cultivated in Middlebrook 7H9 or 7H10 medium supplemented with 0.4% glycerol, 0.5% bovine serum albumin, 0.4% glucose, and 0.05% Tween 80. When required, kanamycin (20 µg/mL) or hygromycin (50 µg/mL) was added to the media. All cultures were incubated at 37 °C. Liquid cultures were grown with agitation at 200 rpm and harvested during the exponential growth phase (Optical Density (OD)_600_ = 0.3–0.6). Plates containing solid cultures were sealed with electrical insulating tape and incubated at 37 °C.

### 4.3. Isolation of Peripheral Blood Mononuclear Cells (PBMCs) and Macrophage Differentiation

Blood samples were collected from the jugular vein of crossbred adult animals (N = 5–7) from INTA bTB free cattle herd following the protocol approved by the Institutional Committee for the Care and Use of Experimental Animals, CICUAE-INTA, under approval number Bo 1. PBMCs were isolated from 50 mL of whole blood per animal using density-gradient centrifugation with Ficoll–Paque, following a standard protocol. Isolated PBMCs were resuspended in RPMI 1640 supplemented with L-glutamine (2 mM), HEPES (25 mM)(Gibco, Thermo Fisher Scientific, Grand Island, NY, USA. cat. 22400089), Antibiotic-Antimycotic (Thermo Fisher Scientific), and 10% autologous plasma, and then seeded into 12-well culture plates at a density of 1 × 10^7^ cells per well. After 24 h, the culture medium was renewed with fresh RPMI and 10% autologous plasma and non-adherent cells were removed by washing with warm phosphate-buffered saline (PBS). Adherent monocytes were maintained in culture for an additional 4 days to allow differentiation into macrophages.

### 4.4. Macrophage Infection

Differentiated macrophages were infected with the different bacterial strains used in this study at a multiplicity of infection (MOI) of 1:1. Each bacterial strain was washed and resuspended in sterile 1× PBS. The suspensions were passed 15 times through a tuberculin needle to disaggregate clumps, followed by centrifugation at 0.2× *g* for 3 min to remove any remaining aggregates. The supernatant containing the isolated bacteria was collected, and its OD_600nm_ was measured to adjust the inoculum to the desired concentration. The infection inoculum was then prepared in RPMI 1640 supplemented with L-glutamine (2 mM) and HEPES (25 mM), without antibiotics and without autologous plasma. For each animal, a non-infected control condition was established. Following a 3 h infection period, cells were washed twice with warm PBS to remove extracellular bacteria. After washing, the culture medium was replaced with fresh RPMI 1640 supplemented with L-glutamine (2 mM), HEPES (25 mM) and 10% autologous plasma. Infected macrophages were then incubated for 16 h prior to RNA isolation.

### 4.5. RNA Extraction and cDNA Synthesis

Total RNA was extracted from macrophages using 500 µL of TRIzol (Invitrogen, Thermo Fisher Scientific, Waltham, MA, USA) reagent per well, according to the manufacturer’s protocol. The quality and quantity of the extracted RNA were assessed by electrophoresis on a 0.8% agarose gel, and by spectrophotometry using a NanoDrop ND-1000 spectrophotometer (Thermo Fisher Scientific, Wilmington, DE, USA). To eliminate genomic DNA contamination, purified RNA was treated with Turbo DNase (Invitrogen, Thermo Fisher Scientific, Waltham, MA, USA). cDNA was synthesized from 500 ng to 1 µg of total RNA per reaction using MML-V reverse transcriptase (Promega, Madison, WI, USA) and 300 ng of random primers (Invitrogen, Thermo Fisher Scientific, Waltham, MA, USA).

### 4.6. Quantitative PCR (qPCR)

qPCR amplification was performed on a StepOne Plus Real-Time PCR System (Applied Biosystems, Foster City, CA, USA) in technical duplicates, using the following cycling conditions: an initial incubation at 95 °C for 5 min, followed by 40 cycles of 60 °C for 30 s and 72 °C for 30 s. Reactions were prepared using the SsoFast EvaGreen Supermix (BIO-RAD, Hercules, CA, USA). GAPDH was used as the reference gene, and the uninfected condition from each corresponding animal served as the calibrator sample. Amplification curves were analyzed with LinRegPCR software (https://www.gear-genomics.com/rdml-tools/ (accessed on 10 November 2025)) [43] to determine cycle threshold (Ct) values and individual reaction efficiencies. Relative gene expression, expressed as fold change, was calculated using the ΔΔCt method with efficiency correction, as implemented in the fg statistical software (https://www.infostat.com.ar/index.php?mod=page&id=34 (accessed on 10 November 2025)). The sequences of all primers used are listed in Table S2.

### 4.7. Two-Hybrid System

*esxB* gene was PCR-amplified with primers LOW_CFP10_PKT25/PUT18C (TCTAGATCAGAAGCCCATTTGCGA) and UP_CFP10_PKT25/PUT18C (CTGCAGGGGCAGAGATGAAGACCGAT) from the Mb04-303 strain and cloned in frame as a fusion to the T25 or T18 fragments of adenylate cyclase by insertion into the *XbaI*/*PstI* sites of the bait (pKT25) and prey (pUT18C) vectors [35]. Proper insertion and sequence integrity were confirmed by DNA sequencing. pKT25 and pUT18C recombinant plasmids carrying either the two alleles of *esxA* (wild type and T63A mutant) were previously obtained [21]. Recombinant bait and prey plasmids were co-transformed into *E. coli* BTH101. Transformants were selected on LB broth supplemented with ampicillin (100 μg/mL) and kanamycin (50 μg/mL) at 30 °C with agitation. Empty pK25 and pUT18C were also used to transform *E. coli* BTH101 and to generate negative control.

β-galactosidase activity was determined in recombinant *E. coli* BTH101 cultures grown at 30 °C for 5 days. Three milliliters of each culture were harvested by centrifugation and resuspended in 1 mL of Z buffer (60 mM Na_2_HPO_4_, 40 mM NaH_2_PO_4_·H_2_O, 10 mM KCl, and 1 mM MgSO_4_·7H_2_O (pH 7.0) plus 50 mM β-mercaptoethanol). Aliquots of 180 µL from each sample were dispensed in triplicate into a 96-well plate. One drop of chloroform was added to each well, followed by 20 µL of o-nitrophenyl β-d-galactoside (ONPG) (4 mg/mL). The plate was incubated at room temperature for 10 min, after which the reaction was stopped by adding 50 µL of 1 M Na_2_CO_3_. Absorbance at 420 nm was then measured using a microplate reader. Cultures resuspended in Z buffer were additionally disrupted using a bead beater (3 cycles of 20 s each at the highest speed) and centrifuged. Total protein content in the supernatants was determined using the Bradford method. Protein concentration was measured at OD_595nm_, and these values were used to normalize β-galactosidase activity measured at OD_420nm_. Data were analyzed with Anova and Tukey’s Multiple Comparison Test.

### 4.8. In Silico Analysis of the T63A Mutation in ESAT-6

The T63A mutation in ESAT-6 was analyzed using computational tools based on the crystal structure of the ESAT-6/CFP-10 complex from *M. tuberculosis* (PDB ID: 3FAV) [ref: PDB DOI: https://doi.org/10.2210/pdb3FAV/pdb (accessed on 21 April 2026)]. The three-dimensional model of the T63A mutant was generated using the BuildModel module of FoldX as implemented in MutaBind2 [44]. Structural visualization and superposition of wild-type and mutant models were performed using UCSF ChimeraX. The root mean square deviation (RMSD) was calculated over Cα atoms of ESAT-6 (residues 2–95). Short-range interprotein contacts between ESAT-6 and CFP-10 were analyzed using a 4 Å distance cutoff.

Protein–protein interaction analysis: MutaBind2 [44] was used to predict the effect of the T63A substitution on ESAT-6/CFP-10 binding affinity. This tool employs a composite scoring function that integrates changes in van der Waals interactions, solvation energy, and evolutionary conservation. The mutation was classified as interfacial or non-interfacial based on its spatial location relative to the binding interface. Default parameters were used, including a 4 Å distance cutoff for contact definition. A mutation was considered non-deleterious if |ΔΔG_bind| < 1.5 kcal/mol, following the tool’s established guidelines.

Protein stability predictions: Three complementary approaches were employed. FoldX (via the BuildModel module within MutaBind2) estimated changes in folding free energy (ΔΔG_fold) calculated on the protein complex. I-Mutant 2.0 [45] was run in structure-based mode, requiring the PDB code (3FAV), chain identifier (B), residue position (63), temperature (25 °C), and pH (7.0) as inputs. PremPS [46] was used with de-fault parameters, providing additional metrics including location (surface or buried), Position-Specific Scoring Matrix (PSSM) value, and solvent-accessible surface area (SASA_pro).

Flexibility simulations: Coarse-grained molecular dynamics simulations were performed using CABS-flex 3.0 to assess backbone fluctuations. Simulations were run in Flexible mode with default parameters (temperature = 1.4, number of cycles = 50, cycles between trajectory frames = 150). Residue-wise root mean square fluctuation (RMSF) profiles were generated for wild-type and T63A complexes to compare dynamic behavior.

### 4.9. Lipid Extraction and Analysis

*M. bovis* strains were cultured to mid-exponential phase (OD_600nm_ ≈ 0.3–0.6) under the conditions described above. For lipid extraction, 50 mL of each culture was collected by centrifugation and resuspended in 1 mL of ultrapure water in pre-weighed 2 mL tubes. Bacterial suspensions were heat-inactivated at 80 °C for 30 min, frozen, and subsequently lyophilized. The dried biomass was weighed to determine the dry weight of each sample prior to lipid extraction.

Total lipids were isolated using a sequential organic solvent extraction procedure adapted from previously described methods. Briefly, dried bacterial pellets were subjected to overnight extraction with chloroform:methanol (1:2, *v*/*v*). The organic phase was recovered, and the residual material was re-extracted twice with chloroform:methanol (2:1, *v*/*v*), each step performed overnight. The final extraction step was performed using chloroform:methanol:water (3:47:48) solution. The lipids present in the final organic phase were dried and quantified by weight. For comparative analyses, lipid samples were resuspended in chloroform:methanol (2:1, *v*/*v*) at volumes normalized according to dry weight to ensure equivalent concentrations among strains.

Total lipid extracts were separated by TLC on silica gel 60 F254 plates, loading equal amounts per lane (300 µg when indicated). PDIMs lipids were resolved using petroleum ether: ethyl acetate (98:2), run three times.

Lipid species were visualized by spraying plates with CuSO_4_–phosphoric acid reagent followed by heating until bands became visible. The experiment was performed in two independent biological replicates.

### 4.10. Statistical Analyses

GraphPad Prism (version 5.0; GraphPad Software, Inc., La Jolla, CA, USA) was used for all statistical analyses and figure generation, except for the pairwise fixed reallocation randomization test. This specific test was performed using fg statistical software.

## 5. Conclusions

Taken together, the combined evidence from *M. tuberculosis* and *M. bovis* studies indicates that ESAT-6 and CFP-10 play complex and context-dependent roles in inflammasome regulation. Their contribution to IL-1β maturation and inflammatory cell death may be shaped by mycobacterial genetic background, host species, and cellular regulatory networks, underscoring the multifaceted nature of ESX-1–mediated host–pathogen interactions.

## Data Availability

The original contributions presented in this study are included in the article/Supplementary Material. Further inquiries can be directed at the corresponding author.

## Supplementary Materials

The following supporting information can be downloaded at: https://www.mdpi.com/article/10.3390/ijms27094099/s1.
