# Supplementary material for: Mycobacterium bovis Strain-Dependent Effects of ESAT-6 and CFP-10 on Inflammasome Activation in Bovine Macrophages
Source: Int J Mol Sci. 2026 May 3;27(9):4099. doi: 10.3390/ijms27094099 (PMC13163879; doi:10.3390/ijms27094099)
Supplement: Supplementary file 1 [file ijms-27-04099-s001.zip › ijms-4235274-supplementary.pdf]

Table S1. In silico prediction of the effect of the T63A mutation on ESAT-6 stability and interaction with CFP-10.

Category	Predictor / Tool	Parameter	Value	Interpretation
**Protein–Protein Interaction (PPI)**	MutaBind2	ΔΔGbind (kcal/mol)	0.01	Neutral to very weak destabilizing effect on protein–protein interaction
	MutaBind2	Interface	No	Mutation is not located at the ESAT-6/CFP-10 interaction interface
**Protein stability and structural context**	FoldX (BuildModel)	ΔΔGfold (kcal/mol)	−0.29	Slight stabilization of the mutant protein compared to wild-type
	PremPS	Stability score	0.21	Low to moderate impact on protein stability
	PremPS	Location	SUR	Mutation located on protein surface
	PremPS	PSSM	0.0427	Low evolutionary conservation
	PremPS	DCS	−0.261	Decrease in conservation upon mutation
	PremPS	SASA_pro	0.102	Consistent with a surface-exposed residue
	I-Mutant 2.0	ΔΔG (kcal/mol)	−0.85	Moderate decrease in intrinsic stability
	I-Mutant 2.0	RSA (%)	55.5	Moderately exposed residue

Table S1. Computational prediction of the impact of the T63A mutation on ESAT-6 stability and interaction with CFP-10 using multiple in silico approaches. All analyses were performed using the same crystal structure (PDB ID: 3FAV). The table summarizes predicted effects on protein–protein interaction stability, intrinsic protein stability, and structural context. Differences in numerical values and sign conventions among predictors reflect algorithm-specific scoring functions and parameters definitions. ΔΔGbind: Predicted change in protein–protein binding free energy (ΔG_mut − ΔG_wt); ΔΔGfold: Predicted change in protein folding stability; PSSM: Position-specific scoring matrix value reflecting evolutionary conservation; DCS: Difference in conservation score between wild-type and mutant residues; RSA: Relative solvent accessibility; SUR: Surface-exposed residue. SASA_pro: Solvent-accessible surface area considering protein atoms only.

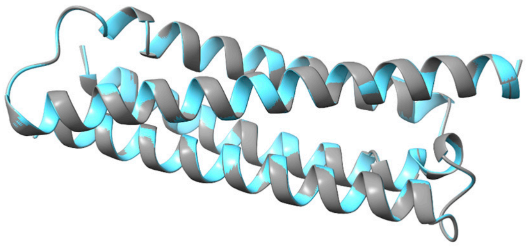

Figure S1. **Structural alignment of the ESAT-6/CFP-10 heterodimer based on the crystal structure (PDB ID: 3FAV) from *M. tuberculosis* and the T63A mutant model (mutaBin2).** The alignment was performed using the Needleman–Wunsch algorithm with the BLOSUM-62 similarity matrix. Secondary structure weighting (SS fraction) was set to 0.3, with gap penalties of 18/18/6 (open) and 1 (extend). The alignment yielded a sequence alignment score of 380.2. The structural superposition shows a root-mean-square deviation (RMSD) approximately 0.1 Å over 74 aligned atom pairs, indicating a high degree of structural similarity.

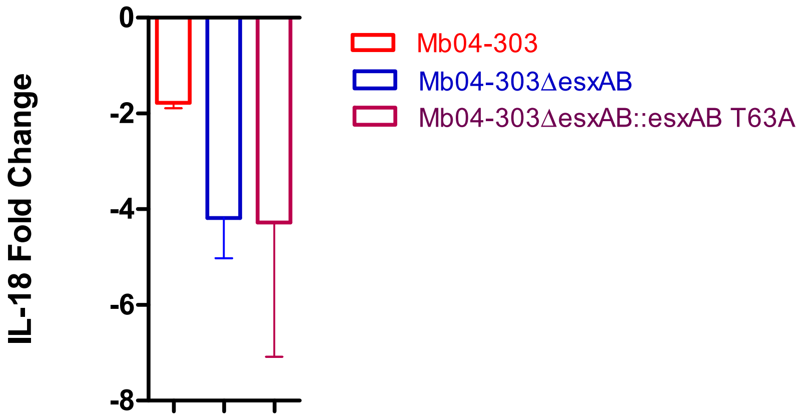

Figure S2. Relative expression of IL-18 in Mb04-303, Mb04-303ΔesxAB and Mb04-303ΔesxAB::esxAB T63A infected macrophages. Macrophages were isolated from 6-7 different cattle and infected at a multiplicity of infection (MOI) of 1:1. Transcript levels were quantified by real-time RT-PCR, using GAPDH as an endogenous reference and uninfected cells as the calibrator sample. Data were analyzed for statistical significance using Kruskal-Wallis test followed by Dunn's post-hoc test for multiple comparisons ($p>0.05$). The Pair Wise Fixed Reallocation Randomization Test was used to assess differences between each infected group and the uninfected control ($p>0.05$).

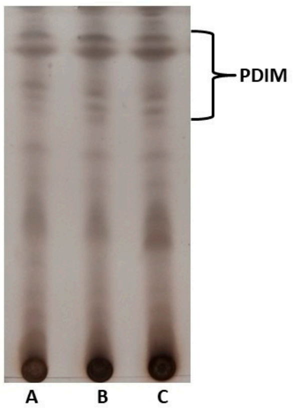

Figure S3. PDIM lipid profiles of different *M. bovis* strains TLC analysis of PDIM from *M. bovis* strains (A) Mb 04-303, (B) Mb 04-303ΔesxAB, (C) Mb 04-303ΔesxAB::esxAB. 300 µg of lipid extracts form each strain were applied to silica gel plates and resolved using petroleum ether and ethyl acetate (98:2, three times).

Table S2. Primers used in RT-qPCR.

Primer	*Sequence*
GAPDH Fwd	ATCTCTGCACCTTCTGCCGA
GAPDH Rev	GCAGGAGGCATTGCTGACA
IL-1β Fwd	TGACCTGAGGAGCATCCTTT
IL-1β Rev	CCAGGGATTTTTGCTCTCTG
AIM2 Fwd	CCCTGCATCCTGAATAGTCC
AIM2 Rev	GGCTTGCATTTTCATGGTTT
NLRP3 Fwd	TCATCACCACAAGACCCGTG
NLRP3 Rev	ATTCCTTCCTCCTGGCCTCT
IL-15 Fwd	GCAGTGCTTTCTCCTGGAGT
IL-15 Rev	TCCTCACATTCTTTGCATCCCA
IL-18 Fwd	GGGCTGCCGTCTTCTGTAA
IL-18 Rev	TCTTCTACTTGTTCTGCAGCCA
